# Detection Method of Epileptic Seizures Using a Neural Network Model Based on Multimodal Dual-Stream Networks

**DOI:** 10.3390/s24113360

**Published:** 2024-05-24

**Authors:** Baiyang Wang, Yidong Xu, Siyu Peng, Hongjun Wang, Fang Li

**Affiliations:** 1 School of Information Science and Engineering, Shandong University, Qingdao 266237, China; 202320482@mail.sdu.edu.cn (B.W.);; 2 School of Information Engineering, Changji University, Changji Hui Autonomous Prefecture, Changji 831100, China

**Keywords:** EEG signal, convolutional neural network, epilepsy diagnosis, feature extraction, classification and diagnosis

## Abstract

Epilepsy is a common neurological disorder, and its diagnosis mainly relies on the analysis of electroencephalogram (EEG) signals. However, the raw EEG signals contain limited recognizable features, and in order to increase the recognizable features in the input of the network, the differential features of the signals, the amplitude spectrum and the phase spectrum in the frequency domain are extracted to form a two-dimensional feature vector. In order to solve the problem of recognizing multimodal features, a neural network model based on a multimodal dual-stream network is proposed, which uses a mixture of one-dimensional convolution, two-dimensional convolution and LSTM neural networks to extract the spatial features of the EEG two-dimensional vectors and the temporal features of the signals, respectively, and combines the advantages of the two networks, using the hybrid neural network to extract both the temporal and spatial features of the signals at the same time. In addition, a channel attention module was used to focus the model on features related to seizures. Finally, multiple sets of experiments were conducted on the Bonn and New Delhi data sets, and the highest accuracy rates of 99.69% and 97.5% were obtained on the test set, respectively, verifying the superiority of the proposed model in the task of epileptic seizure detection.

## 1. Introduction

Epilepsy is a common neurological disorder characterized by abnormal electrical activity in the brain. These abnormal electrical activities can trigger various forms of seizures, which vary from person to person. Seizures may cause generalized convulsions, which are violent, involuntary contractions and spasms of muscles throughout the body. This type of seizure is often called a generalized seizure. However, epileptic seizures do not always manifest as generalized convulsions. Some types of seizures, such as focal seizures, may be limited to one part of the body and manifest as localized muscle twitching or abnormal sensations. In addition, some seizures may include brief loss of consciousness, abnormal behavior, or confusion without obvious convulsions. Epilepsy can manifest in many different ways, depending on where in the brain the abnormal electrical activity occurs and how it spreads [1]. The diagnosis and monitoring of epilepsy relies heavily on electroencephalography (EEG), a non-invasive brain testing technique that measures electrical signals in the brain through electrodes attached to the scalp. EEG can capture changes in brain activity during seizures. However, EEG signals are complex, noisy, and high-dimensional, which poses a challenge for accurate and efficient classification of EEG signals [2].

EEG signals for the detection and diagnosis of epilepsy can be divided into the following steps: signal preprocessing, feature extraction, feature selection and classification [3]. Signal preprocessing is carried out to remove noise and interference from the EEG signal and improve the signal quality. Then, useful features are extracted from the EEG signal that reflect the time domain, frequency domain, or time–frequency domain characteristics of the signal. However, not all of the extracted features are favorable for epilepsy diagnosis, so feature selection is also needed to select the optimal subset from the extracted features to reduce the feature dimensionality and computational complexity. Finally, the EEG signals are categorized as either normal or abnormal based on the features, which also help to further differentiate the type and degree of epilepsy. In recent years, many researchers have proposed many intelligent epilepsy diagnostic methods, some of which are based on traditional signal processing methods, and some of which are based on machine learning and deep learning methods.

Conventional methods usually require an artificially designed approach to feature extraction and selection [4,5,6,7,8,9,10,11]. Guangpeng et al. [12] extracted the time–frequency feature maps of interval EEG signals. Then a single-channel method was used to reduce the network parameters, and finally a convolutional neural network was used to predict epilepsy, with a prediction accuracy of 87.9%. Cansel et al. [13] used discrete wavelet transform to process EEG signals and thus diagnose temporal lobe epilepsy (TLE) patients and psychogenic nonepileptic seizure (PNES) in an automated discriminative method to quickly and accurately determine different epilepsy types. Sirin et al. [14] investigated the interaction between sleep architecture and seizure probability, using dual-channel subcutaneous EEG signals to account for changes in brain dynamics in each patient. Seyed Morteza et al. [15] also used discrete wavelet transform to decompose the EEG signal; however, it was based on the Modified Binary Salp Swarm Algorithm (MBSSA) to extract the time domain features, thus avoiding manual and time-consuming computations. Ying et al. [16] used a wearable EEG monitoring device to capture EEG and automated epilepsy detection using support vector machines, providing a new approach to real-time monitoring. Traditional methods have certain limitations and need to set up appropriate recognition methods according to specific scenarios, which is not conducive to the rapid diagnosis of epilepsy diseases for complex and variable epilepsy types [17,18,19].

Deep learning methods can automatically learn feature representations from raw signals. Deep learning methods can also process multi-channel EEG signals, utilizing the spatial relationships between the signals [20,21,22,23,24]. Zixu et al. [25] developed a unified framework early-seizure detection and epilepsy diagnosis using mainly autoregressive moving average-model and support-vector machine classifiers for epilepsy diagnosis, which achieved classification accuracies of 93% and 94%, respectively. Weidon et al. [26] used multichannel EEG signals to construct a multilayer deep convolutional neural network model, thus effectively utilizing the relevant information such as time, frequency, and channel of EEG to extract relevant features about epilepsy, which greatly improved the diagnostic accuracy of epilepsy. Abdelhamid et al. [27] proposed a framework combining deep learning and EEG signal processing without any manual feature extraction for the detection of seizures and non-seizures, with a combination of one-dimensional convolutional neural networks, recurrent neural networks, and attentional mechanisms, which achieved high recognition accuracy in several publicly available datasets. Mingyang et al. [28] proposed a neural network based on wavelet envelope analysis, which combines discrete wavelet transform with the envelope analysis method to extract important features from EEG signals. Aayesh et al. [23] performed time domain, frequency domain and nonlinear analysis on the signal to extract pattern features; they performed feature selection on the extracted features, obtained more discriminative features, and constructed a fuzzy machine learning classifier for epileptic seizure detection.

Combining these methods, for the feature extraction of EEG signals, in this study we used the differential features of the EEG signal, the amplitude spectrum and the phase spectrum to jointly extract the features of the EEG signal. Differential feature extraction is a commonly used signal processing method that captures changes in a signal by calculating the difference between consecutive time points. In EEG signal processing, the change trend of the signal can be obtained by calculating the difference between adjacent time points, thereby extracting the differential features and capturing the instantaneous or periodic changes in the signal. Frequency domain analysis is the process of converting signals from the time domain to the frequency domain. The amplitude spectrum represents the amplitude of the signal at different frequencies, while the phase spectrum represents the phase information of the signal at different frequencies. In EEG signal processing, frequency domain analysis can help reveal the different frequency components present in the signal, such as alpha waves, beta waves, etc., as well as the phase relationship between them, and help identify activity patterns at specific frequencies in the signal, thereby better understanding and analyzing the characteristics of EEG signals. Differential feature extraction helps capture the instantaneous changes in the signal, while the amplitude spectrum and phase spectrum in the frequency domain provide information about the amplitude and phase of the signal at different frequencies. The combined use of these methods can more comprehensively describe the characteristics of EEG signals. This provides richer feature information for subsequent signal analysis and processing.

The preprocessed data uses a neural network model based on a multi-modal dual-stream network to process temporal features and spatial features, respectively. Specifically, it is divided into two streams, one for processing temporal features and the other for processing spatial features. The two streams can each adopt network structures and algorithms suitable for processing their respective characteristics, with improved processing and representation capabilities of complex signals.

The remainder of this article is organized as follows. Section 2 introduces the EEG dataset and methods. Section 3 describes the experimental procedure and results. Section 4 concludes the paper and suggests some future directions.

## 2. EEG Data Sets and Methods

### 2.1. Dataset

#### 2.1.1. The University of Bonn Dataset

**Figure 1:**
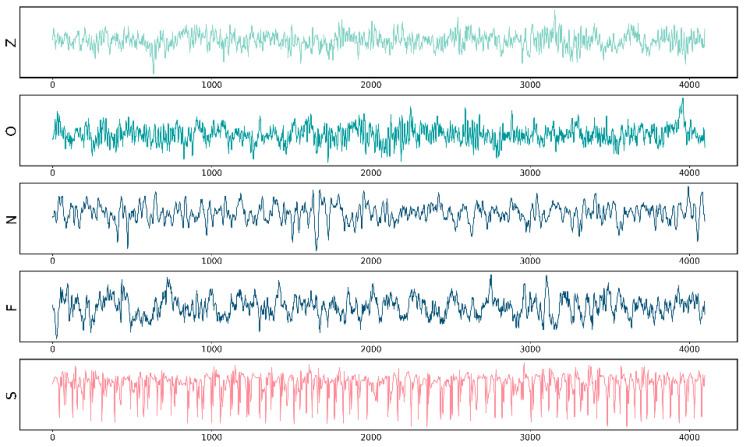
Bonn EEG Dataset EEG visualization.

Bonn EEG Dataset is one of the public data sets widely used in the field of brain–computer interface (BCI) and neuroscience research [29]. The dataset was created by the Center for Medical Epilepsy at the University of Bonn in Germany. This data set contains EEG data from 5 healthy people and 5 epilepsy patients. It was collected using the international 10–20 system EEG acquisition system. It contains a total of 5 data subsets, namely F, S, N, Z, and O. The data are described in Table 1 and visualized in Figure 1. The Bonn data set is a single-channel data set, in which each sub-data set contains 100 data segments: the time length of each data segment is 23.6 s, the data points are 4097, and the sampling frequency is 173.61 Hz.

Subsets Z and O were collected from a control group of 5 healthy individuals. The clip in Z is the EEG when the subject’s eyes are open, and the clip in O is the EEG when the subject’s eyes are closed. Subsets N, F, and S are intracranial EEG, collected from 5 patients who were diagnosed before surgery. Subset N comes from the intracranial hippocampal formation area of the patient’s interictal period. Subset N comes from the intracranial hippocampal formation area of the patient’s interictal period. Subset F comes from the intracranial lesion area of the patient during the interictal period. Subset S comes from the intracranial lesion area during the patient’s ictal period. In the experiment, Z, O, N, and F are regarded as one category and marked as Interictal period. E is marked Ictal period. Slice the data into a 2 s time window to obtain a single training sample.

#### 2.1.2. New Delhi Dataset

**Figure 2:**
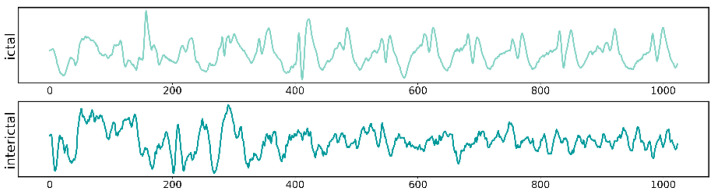
New Delhi EEG Dataset EEG Visualization.

The New Delhi dataset is a publicly available dataset created from the Center for Neurology and Sleep, Hauz Khas, New Delhi. The dataset contains EEG recordings of ten epilepsy patients [30]. Data were collected using a Grass Telefactor Comet AS40 amplification system at a sampling rate of 200 Hz. During the acquisition process, gold-coated scalp EEG electrodes were placed according to the 10–20 electrode placement system. The signal is filtered between 0.5 and 70 Hz and then divided into pre-ictal, interictal and ictal. Each category contains MAT files of 50 EEG time-series signals. The sampling frequency is 200 Hz, and each MAT file contains 1024 samples. Each sample represents a set of EEG time-series data with a duration of 5.12 s. The EEG signal is shown in Figure 2.

### 2.2. Data Set Preprocessing

The EEG signal reflects the activity process of the brain. The amplitude of the EEG signal changes within the entire range of 2~100 μV, and the frequency range is 1~100 Hz. In the study, the EEG was divided into five frequency sub bands. In general, delta waves often appear in the cerebral cortex during deep sleep. Specifically, this electrical activity brain waveform with a frequency between 0.5 and 4 Hz is consistent with the deepest stage of non-rapid eye movement sleep, and is associated with an extremely relaxed and restorative state of the brain and body. In contrast, theta waves, with frequencies between 4 and 8 Hz, usually appear in the shallow stages of sleep and during meditation, reflecting a transitional state between wakefulness and sleep, involving memory and learning process. Alpha waves, with a frequency between 8 and 12 Hz, are clearly present in the cerebral cortex when a person is not stressed and calm, especially in the occipital area. This waveform is most significant when resting with eyes closed or lightly relaxed, marking a state of being awake but relaxed. The frequency of beta waves is between 12 and 30 Hz. It generally appears when the frontal lobe is excited and thinking. It is related to active cognitive activities and high concentration. It is commonly seen in problem solving, decision-making and reasoning processes. Finally, gamma waves, with frequencies above 30 Hz, typically occur when the brain feels anxious or in a state of emotional stress. Although this waveform is associated with high levels of cognitive function and information processing, in states of stress or anxiety, gamma wave activity also increases significantly [31,32,33].

In this study, in order to better observe the different EEG signal characteristics of patients, a signal is first converted from the time domain to the frequency domain. Its Fourier-transformed $x_{1}$ is the representation of the signal in the frequency domain, which contains the signal amplitude and phase information of x. The amplitude of the signal in the frequency domain is then calculated. By taking the absolute value of the Fourier transform result $x_{1}$, we obtain the amplitude spectrum $x_{2}$ of the signal. By taking the angle of the Fourier transform result $x_{1}$, the phase spectrum $x_{3}$ of the signal is obtained.

**Figure 3:**
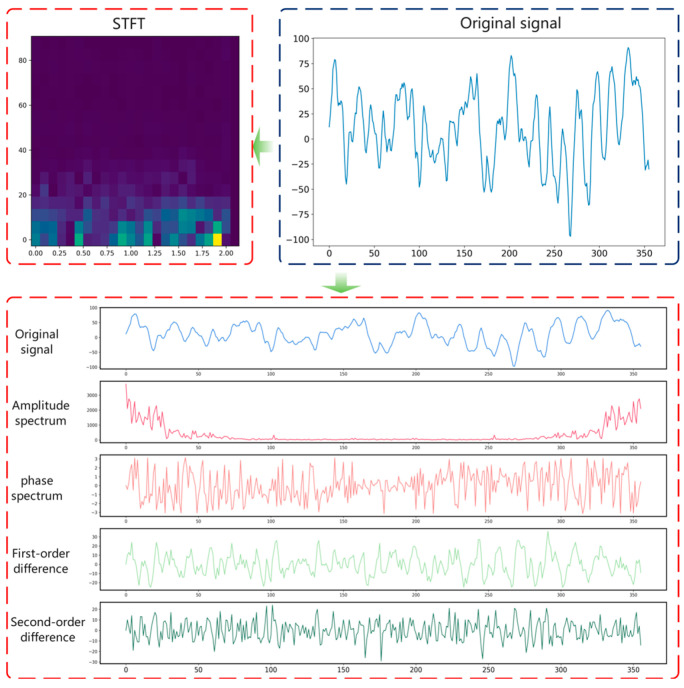
EEG signal processing process.

Finally, calculate the first-order difference $x_{4}$ and the second-order difference $x_{5}$ of the signal x. Finally, a feature matrix $\left[x,\;x_{2},\;x_{3},\;x_{4},\;x_{5}\right]$ is formed with the original signal. On the other hand, the short-time Fourier transform is performed on the original signal to obtain spectrum data $x_{6}$, which contain the signal at different frequencies. The EEG processing flow is shown in Figure 3.

#### 2.2.1. FFT (Fast Fourier Transform) and (Short-Time Fourier Transform) STFT

FFT functions to calculate the Discrete Fourier Transform (DFT) of the input signal $x_{0}$ [34,35,36]. It converts a signal from the time domain to the frequency domain and represents the signal as a collection of frequency components. The discrete form of DFT can be expressed as Formula (1), where x[n] is the discrete sample of the input signal, X[k] is the transformed signal, N is the number of samples of the signal, and i is the imaginary unit.

(1) $$X[k]=\sum_{n=0}^{N-1}x\left[n\right]\times e^{-2\pi i\cdot \frac{kn}{N}}$$

STFT decomposes the signal into two dimensions: time and frequency. It segments the signal in time and applies Fourier transform to each time segment to obtain the representation of the signal in frequency [37]. The specific principle formula is as follows: Formula (2), where X(t,ω) is the STFT result of the time domain signal  x(t) at frequency ω, w(τ−t) is the window function, usually using the Hanning window and other window functions, and ω is the angular frequency.

(2) $$X(t,\omega )=\int_{-\infty }^{\infty }x(\tau )\cdot w(\tau -t)\cdot e^{-j\omega \tau }d\tau$$

STFT is usually implemented through discretization, replacing continuous time and frequency with discrete time and frequency. For discrete signals, STFT can be expressed as Formula (3), where X[m,ω] is the STFT result of the discrete time-domain signal x[n] at frequency ω, w[n−m] is the discrete window function, and m is the time index.

(3) $$X[m,\omega ]=\sum_{n=-\infty }^{\infty }x[n]\cdot w[n-m]\cdot e^{-j\omega n}$$

#### 2.2.2. First-Order Difference and Second-Order Difference

The difference operation refers to calculating the difference between each element in the array and the adjacent element to obtain a new array. When the calculation result is the difference between the current data point and the next data point, it is called the forward difference. The calculation result is a positive value, which means that the function is rising at that point; if it is a negative value, it means that the function is falling at that point. The formula for directional difference is shown as Formula (4).

(4) $$\Delta^{2}f(x)=f(x+1)-f(x)$$

When the calculation result is the difference between the current data point and the previous data point, it is called backward difference. When the calculated result is positive, it means that the function is rising at that point. If it is negative, it means that the function decreases at that point. The principle is shown in Formula (5).

(5) $$\nabla^{2}f(x)=f(x)-f(x-1)$$

First difference refers to the operation of calculating the difference between each element in a sequence and its previous element. Second-order difference refers to a new sequence obtained by performing two difference operations on a sequence. The formula of the forward second-order difference is shown in Formula (6).

(6) $$\Delta^{2}f(x)=f(x+2)-2f(x+1)+f(x)$$

The formula for the backward second-order difference is shown in Formula (7).

(7) $$\nabla^{2}f(x)=f(x)-2f(x-1)+f(x-2)$$

For a sequence $\left[a_{1},a_{2}\;,a_{3}\;,...,a_{n}\right]$, its first difference can be expressed as $\left[b_{1},b_{2}\;,b_{3}\;,...,b_{n-1}\right]$, where $b_{i}=a_{i+1}-a_{i};$ then perform a difference operation on the first-order difference sequence, and the result is the second-order difference sequence $\left[c_{1},c_{2}\;,c_{3}\;,...,c_{n-2}\right]$, where $c_{i}=b_{i+1}-b_{i}$.

### 2.3. Neural Network Module

#### 2.3.1. One-Dimensional Convolutional Neural Network

**Figure 4:**
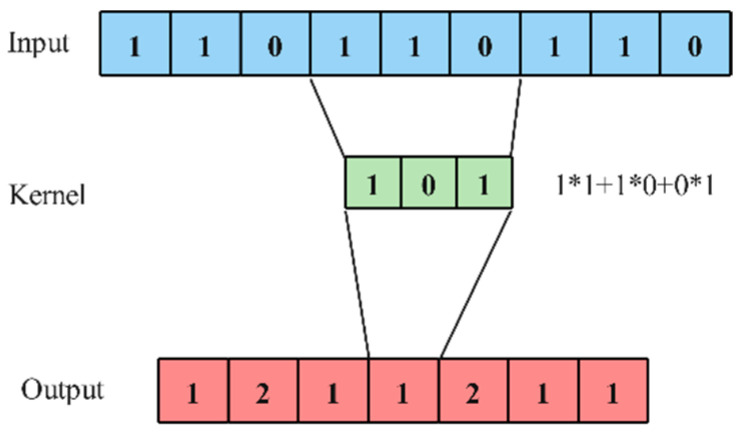
One-dimensional convolution principle.

One-dimensional convolution is often used to process time series data, using a one-dimensional convolution kernel of a specified size to perform a one-dimensional convolution operation on the input multi-channel one-dimensional input signal [38]. Assume that the size of the input is $\left(N,C_{in},L_{in}\right)$, where N represents the batch size, $C_{in}$ represents the number of channels, and $L_{in}$ represents the length of the signal sequence. The size of the output is $\left(N,C_{out},L_{out}\right)$, where $C_{out}$ represents the number of output channels and $L_{out}$ represents the length of the output signal. The operation formula is as shown in Formula (8), and ∗ represents a valid cross-correlation operator. The principle is shown in Figure 4.

(8) $$\mathrm{out}(N_{i},C_{{\mathrm{out}}_{j}})=\mathrm{bias}(C_{{\mathrm{out}}_{j}})+\sum_{k=0}^{C_{\mathrm{in}}-1}\mathrm{weight}\left(C_{{\mathrm{out}}_{j}},k\right)*\mathrm{input}(N_{i},k)$$

#### 2.3.2. Two-Dimensional Convolutional Neural Network

**Figure 5:**
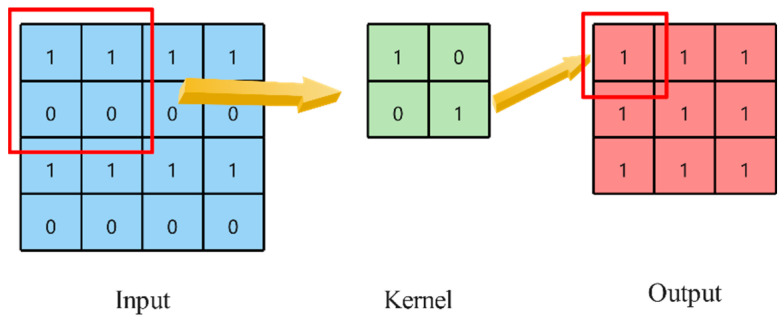
Two-dimensional convolution principle.

Two-dimensional convolutional layers are used to process two-dimensional input signals [39]. Assume that the size of the input is $\left(N,C_{in},H_{in},W_{in}\right)$, where N represents the batch size, $C_{in}$ represents the number of input channels, and $H_{in}$ and $W_{in}$ represent the height and width of the input image, respectively. The size of the output is $\left(N,C_{out},H_{out},W_{out}\right)$, where $C_{out}$ represents the number of output channels, and $H_{out}$ and $W_{out}$ represent the height and width of the output image, respectively. Here, ∗ represents a valid two-dimensional cross-correlation operator. The formula of two-dimensional convolutional is as shown in Formula (9). The principle is shown in Figure 5.

(9) $$\mathrm{out}(N_{i},C_{{\mathrm{out}}_{j}})=\mathrm{bias}(C_{{\mathrm{out}}_{j}})+\sum_{k=0}^{C_{\mathrm{in}}-1}\mathrm{weight}(C_{{\mathrm{out}}_{j}},k)*\mathrm{input}(N_{i},k)$$

#### 2.3.3. Long Short-Term Memory (LSTM)

**Figure 6:**
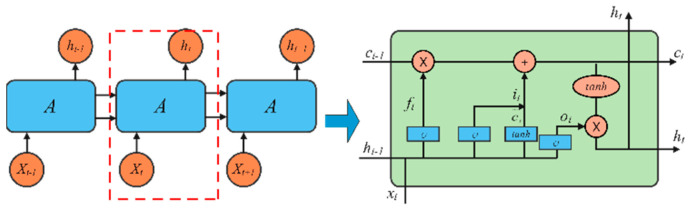
LSTM structure.

LSTM is a special type of RNN. In order to solve the problems of gradient disappearance and gradient explosion that exist in traditional RNN, memory cells and gating mechanisms are introduced, which can retain old feature information in feature extraction of sequence data, thereby extracting relevant features. This achieves a better performance in data feature extraction [40]. Figure 6 below shows the network structure of LSTM.

There are three types of gates in the LSTM gate: input gate i, forget gate f and output gate o. The input gate is used to control the update information of the storage unit. The forget gate is used to control the amount of storage unit information used at the previous moment. The output gate is used to control the amount of information output to the next hidden state. At time t, given the input vector $t_{x}$ and the hidden state $h_{t-1}$ at the previous moment, the LSTM unit calculates the hidden state $h_{t}$ at the current moment through internal loops and updates, and the formula is shown in (10)–(15).

(10) $$f_{t}=\sigma \left(w_{fx}x_{t}+w_{fh}h_{t-1}+b_{f}\right)$$

(11) $$i_{t}=\sigma \left(w_{ix}x_{t}+w_{ih}h_{t-1}+b_{i}\right)$$

(12) $${\tilde{c}}_{t}=\varphi \left(w_{cx}x_{t}+w_{ch}h_{t-1}+b_{c}\right)$$

(13) $$c_{t}=\sigma \left({\tilde{c}}_{t}i_{t}+f_{t}c_{t-1}\right)$$

(14) $$o_{t}=\sigma \left(w_{ox}x_{t}+w_{oh}h_{t-1}+b_{0}\right)$$

(15) $$h_{t}=\varphi \left(c_{t}o_{t}\right)$$

Among them, $w_{fx}$, $w_{ix}$, $w_{cx}$, and $w_{ox}$ represent the weight matrix between the input layer and the corresponding gate at time t. $w_{fh}$, $w_{ih}$, $w_{ch}$, and $w_{oh}$ are the hidden-layer weight matrices between time values t and t−1, and $b_{f}$, $b_{i}$, $b_{c}$, and $b_{o}$ represent the corresponding deviations. ${\;h}_{t-1}$ and $c_{t-1}$ are the hidden state and cell state of time value t−1, and $i_{t}$, $f_{t}$, and $o_{t}$ are the output values of the input gate, forgetting gate and output gate respectively. $c_{t}$ and $h_{t}$ correspond to the cell state and hidden state at the current time t, respectively, $c_{t}$ represents the temporary cell state, and φ and σ represent the tanh and sigmoid activation functions, respectively.

## 3. Methods

### 3.1. Overall Process of Detection Method of Epileptic Seizures Using a Neural Network Model Based on Multimodal Dual-Stream Networks

In order to utilize EEG signals to identify patients with epilepsy, a neural network model based on a multimodal two-stream network was adopted, with a mixed use of one-dimensional convolution, two-dimensional convolution and the LSTM neural network to extract the spatial characteristics of EEG and the temporal characteristics of the signal, respectively. Combining the advantages of the two networks can more comprehensively extract EEG features. This method includes the following steps.

- Data preparation: obtain and prepare Bonn and New Delhi datasets for experiments; these contain EEG signal data on epileptic seizures.
- Feature extraction: Preprocess the original EEG signal, including filtering and noise removal. Extract the differential characteristics of the signal, the amplitude spectrum and the phase spectrum in the frequency domain to form a two-dimensional feature vector.
- Establish a multi-modal dual-stream network model: Design and build a multi-modal dual-stream network model, combining one-dimensional convolution, two-dimensional convolution and the LSTM neural network. The first-class network is used to extract the spatial features of the EEG two-dimensional vector, while the other-stream network focuses on extracting the temporal features of the signal. Utilizing a hybrid neural network structure, temporal and spatial features are simultaneously extracted from signals to enhance recognition performance. A channel attention module is introduced to improve the model’s attention to features related to epileptic seizures.
- Experiment: The Bonn and New Delhi data sets are divided into training sets, validation sets and test sets. Train, validate, and test the model to evaluate its performance. The performance of the model on the epileptic seizure detection task was evaluated using accuracy, recall, precision, and F1 score.
- Result analysis: analyze the experimental results and compare the performance differences between the proposed model and the baseline model.

### 3.2. Neural Network Model Based on Multimodal Dual-Stream Networks

Based on one-dimensional convolution, two-dimensional convolution and LSTM modules, we designed a neural network model of a multi-modal two-stream network to solve the epileptic seizure detection task. The architecture of the model is as follows.

Time-series signal processing flow:

- Input: 5×356 time-series signal, including original signal, first-order difference, second-order difference, amplitude spectrum and phase spectrum in frequency domain.
- Processed through three one-dimensional convolution modules, a 256×356 feature vector $y_{1}$ is output.
- Perform batch normalization and ReLU activation function on $y_{1}$, and then add it to the feature vector $y_{2}$ processed by a one-dimensional convolution module to obtain $y_{3}$.
- Input y1 into the LSTM network to obtain a 356×4  output feature vector $y_{4}$.

STFT matrix processing flow:

- Input: STFT matrix of the original signal.
- Processed through three two-dimensional convolution modules, batch normalization and ReLU activation function, a 256×11×18 feature matrix $y_{5}$ is obtained.

Feature fusion and classification:

- Flatten $y_{3}$, $y_{4}$, and $y_{5}$ and concatenate them into one eigenvector.
- Output the feature vector to the fully connected layer and output the classification probability through softmax.

**Figure 7:**
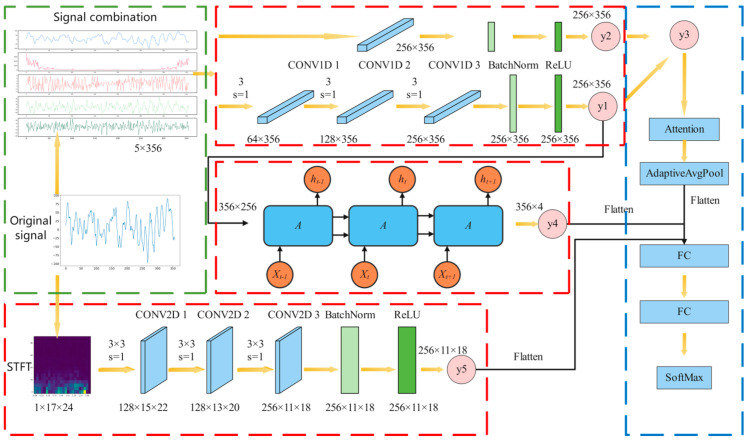
The overall process involved in the detection method for epileptic seizures.

Based on EEG signals, the model fuses features from time series signals and STFT matrices, uses one-dimensional convolution, two-dimensional convolution, and LSTM modules to extract temporal and spatial features, respectively, and performs classification through fully connected layers to achieve automatic epileptic seizure detection. The network structure is shown in Figure 7.

## 4. Experimental Results and Analysis

The hardware devices used in this article are Inter i7 13700k and Nvidia RTX4080, Intel i7-13700K, is sourced from Intel Corporation, which is headquartered in Santa Clara, CA, USA. Nvidia RTX 4080 is sourced from Nvidia Corporation, which is headquartered in Santa Clara, CA, USA. These two devices have high-performance processing capabilities and can meet complex computing needs. The ratio of training set, validation set and test set is set to 8:1:1. The software environment used is Python 3.8. Using accuracy, recall rate, and *F*1-*score* as the evaluation criteria of the model, these three indicators reflect the prediction ability, coverage ability, and comprehensive ability of the model, respectively.

Precision refers to the proportion of samples that are actually positive samples among all the samples that are predicted to be positive. The calculation formula is as shown in Formula (16), where TP represents true examples, TN represents true counterexamples, *FP* represents a false positive example, and FN represents a false negative example.

(16) $$\mathrm{precision}=\frac{\mathrm{TP}}{\mathrm{TP}+\mathrm{FP}}$$

Recall refers to the proportion of samples that are successfully predicted as positive samples among all positive samples. The calculation formula is shown as Formula (17).

(17) $$\mathrm{recall}=\frac{\mathrm{TP}}{\mathrm{TP}+\mathrm{FN}}$$

*F*1-*score* represents the balance between *precision* and *recall*, and the calculation formula is shown as Formula (18).

(18) $$f1-\mathrm{score}=\frac{2\times \mathrm{precision}\times \mathrm{recall}}{\mathrm{precision}+\mathrm{recall}}$$

Use a confusion matrix to place the predicted results and true results of all categories into the same table by category. In this table, there are the number of correct identifications and the number of incorrect identifications for each category. Cluster analysis of data can better observe experimental results, find out the relationship between various categories, and make the data concise. t-SNE technology can reduce the dimensionality of high-dimensional data in the CNN fully connected layer to two dimensions, so that we can intuitively judge the performance of the current model [41].

### 4.1. Bonn Dataset

In the EEG data from the University of Bonn, we treat Z, O, N and F as one category, labeled as the interictal period. The E mark indicates the ictal period. Then, we use the proposed neural network model for training. The experiment is divided into two phases: the training phase and testing phase.

In the training phase, we trained for 30 epochs. Finally, on the validation set, we achieved an accuracy of 99.2% with a loss function of 0.03082. Cross-validation is a method used to observe the stability of the model. We divide the data into n parts, use one part as the test set, one part as the validation set, and the other n − 2 parts as the training set, and calculate multiple times. The accuracy of the model is used to evaluate the average accuracy of the model, as shown in Equation (19).

(19) $$p=\frac{1}{10}\sum_{i=1}^{10}p_{i}$$

where *p* refers to the accuracy obtained by each verification. After cross-validation, the average accuracy decreased slightly, and an accuracy of 98.55% was obtained.

**Figure 8:**
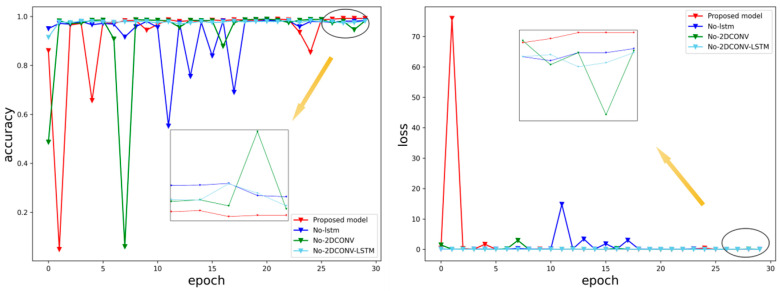
The training accuracy and loss function of the proposed network, as well as the removal of the LSTM module, the removal of the two-dimensional convolution module, and the accuracy and loss functions of the ablation experiment in the Bonn data set by removing the LSTM and two-dimensional convolution module at the same time.

**Figure 9:**
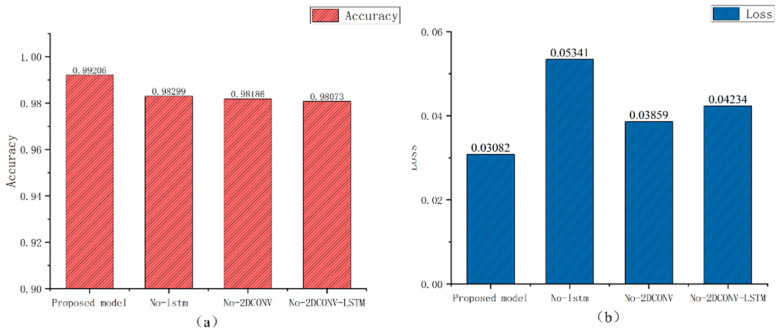
The proposed network removes the LSTM module, removes the two-dimensional convolution module, and simultaneously removes the LSTM and two-dimensional convolution module. Ablation experimental performance on the Bonn dataset: (**a**) highest training accuracy (**b**) loss function.

In addition, we also conducted ablation experiments, removing the LSTM module and two-dimensional convolution module of the network, and removing both the LSTM and two-dimensional convolution modules to verify the effectiveness of the network. The results are shown in Figure 8 and Figure 9.

The results of the training phase:

- Remove the LSTM module: the accuracy is 98.2%, and the loss function is 0.05341.
- Remove the two-dimensional convolution module: the accuracy is 98.1%, and the loss function is 0.03859.
- Remove the LSTM and two-dimensional convolution modules at the same time: the accuracy is 98%, and the loss function is 0.04234.

The combination of multiple modules has the advantage of better extracting the characteristics of EEG signals.

**Figure 10:**
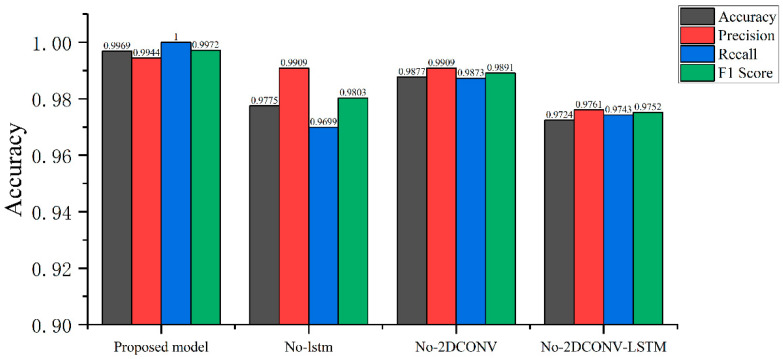
The proposed network removes the LSTM module, removes the two-dimensional convolution module, and simultaneously removes the LSTM and two-dimensional convolution modules in the Bonn test set ablation experiment. *Accuracy*, *precision*, *recall*, and *F*1-*score* are shown.

In the testing phase, the performance of the saved network model was tested using the test set and evaluated using precision, recall, and F1 scores. Finally, on the test set, the accuracy of our proposed network model was 0.9969, precision was 0.9944, recall was 1, and *F*1-*score* was 0.9972. In the ablation experiment, the LSTM module and two-dimensional convolution module of the network were removed respectively, and the accuracy, recall rate, and *F*1-*score* results of removing the LSTM and two-dimensional convolution module at the same time are shown in Figure 10.

- Remove the LSTM module: accuracy 0.9775, *precision* 0.9909, *recall* 0.9699, *F*1-*score* 0.9803.
- Remove the two-dimensional convolution module: *accuracy* 0.9877, *precision* 0.9909, *recall* 0.9873, *F*1-*score* 0.9891.
- Remove the LSTM and two-dimensional convolution modules at the same time: *accuracy* 0.9724, *precision* 0.9761, *recall* 0.9743, *F*1-*score* 0.9752.

In the test set, the neural network combined with one-dimensional convolution, two-dimensional convolution and the LSTM multi-module achieved greater performance advantages in the face of test data that did not appear during the training process, which shows that multi-modal feature extraction is more conducive to improving the generalization ability of the model.

**Figure 11:**
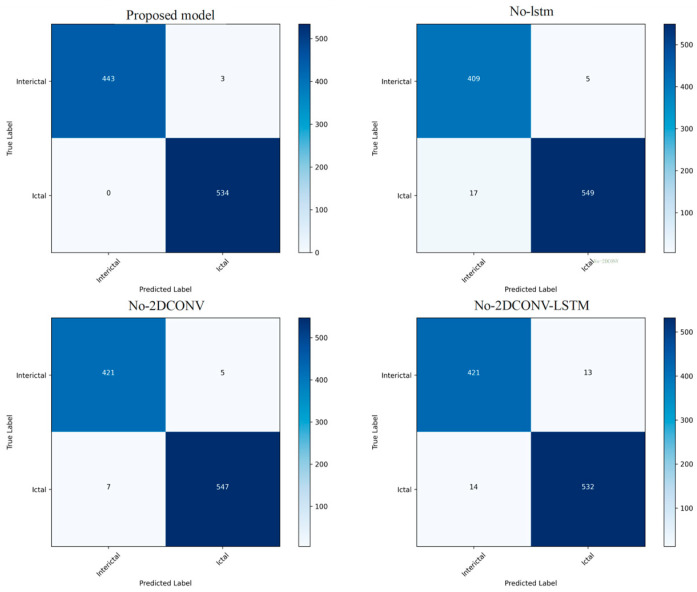
The proposed network, the LSTM module is removed, the two-dimensional convolution module is removed, and the LSTM and two-dimensional convolution modules are removed at the same time, and the confusion matrix of the Bonn test set ablation experiment is shown.

Finally, the confusion matrix and t-SNE are used to visualize the predicted distribution of the test data. Figure 11 contains the confusion matrices of four different models, namely, the proposed model, No-lstm, No-2DCONV and No-2DCONV-LSTM. Each confusion matrix shows the classification results between two categories (interictal and ictal), including true examples, false-positive examples, true-negative examples, and false-negative examples. Each confusion matrix represents different classification situations with different colors, and darker colors represent higher numbers. The model proposed in this article has obtained the best classification effect.

**Figure 12:**
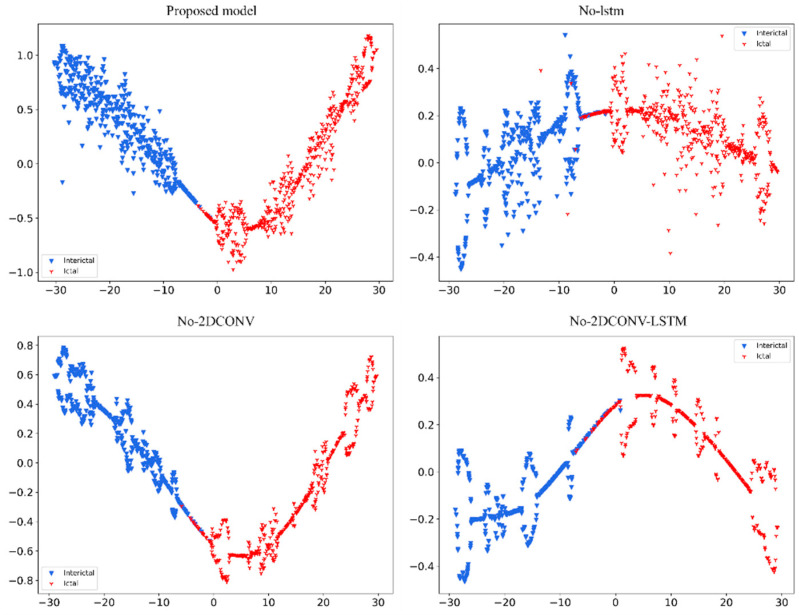
The proposed network removes the LSTM module, removes the two-dimensional convolution module, removes both the LSTM and the two-dimensional convolution module, and performs cluster analysis on the Bonn test set ablation experiment.

Figure 12 of t-SNE shows the clustering of data for four different models. Each subgraph has two colors of points, representing two different types of data. The proposed model has the least confounded classification results between the two categories (interictal and ictal).

### 4.2. New Delhi Dataset

**Figure 13:**
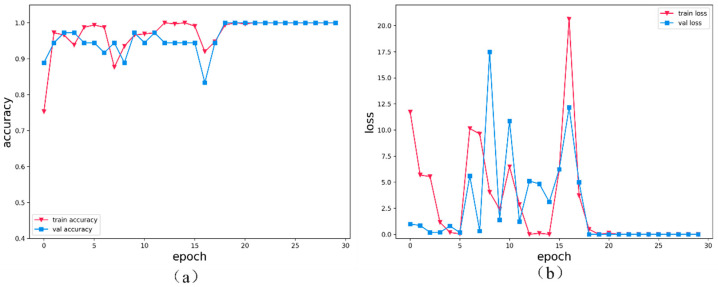
Performance of the proposed network on the New Delhi dataset: (**a**) accuracy (**b**) loss function.

In order to verify the effectiveness and generalization ability of the proposed network, the public New Delhi dataset was used to verify the model performance. In the EEG data of New Delhi, we used two categories of EEG data: interictal and ictal. The experiment is divided into two phases: the training phase and testing phase. In the training phase, 30 epochs were trained. The accuracy and loss functions in the training phase are shown in the figure. The final accuracy is 1 and the loss function is 6.71 × 10^−8^. The results are shown in Figure 13.

**Figure 14:**
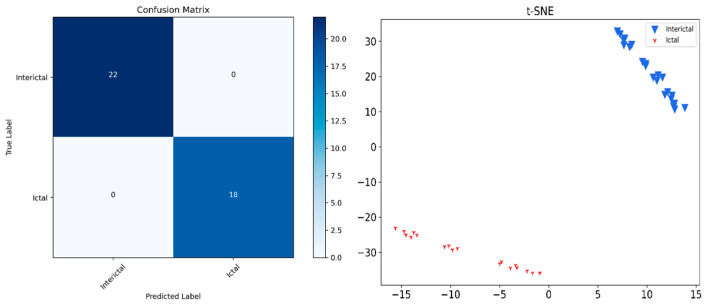
Confusion matrix and cluster analysis of the proposed network on New Delhi dataset.

On the test set, the obtained *accuracy* is 0.975, *precision* is 0.9444, *recall* is 1, and *F*1-*score* is 0.9714. The confusion matrix and cluster analysis are shown in Figure 14. It can be seen that the proposed model still has good accuracy when training and predicting using a new EEG data set without changing the network structure, verifying the improvement in the effectiveness and generalization ability of the model.

### 4.3. Comparison and Discussion with Related Studies

A seizure detection method based on multimodal two-stream networks is proposed and validated using the widely recognized University of Bonn dataset, as shown in Table 2. Compared with the existing methods, the proposed method outperforms the existing methods in all main performance metrics [42]. Richhariya and Tanveer [15] used PCA, ICA and DWT to achieve an accuracy of 99.0%. Li et al.’s [28] method based on wavelet envelope analysis achieved an accuracy of 98.8%. Shen et al. [43] adopted the methods of discrete wavelet transform and support vector machine and achieved an accuracy of 97% and a sensitivity of 96.67%, while Xu et al. [44] used the 1D CNN-LSTM method to improve *accuracy*, *precision*, *recall* and *F*1-*score*, respectively, reaching 99.39%, 98.39%, 98.79% and 98.59%. In contrast, the multi-modal dual-stream network method we proposed achieved an *accuracy* of 99.69%, a *precision* of 99.44%, a *recall* of 99.00%, and an *F*1-*score* of 99.72%. These results show that our method not only achieves the highest value in accuracy, but also significantly outperforms existing methods in key performance indicators such as *precision*, *recall*, and *F*1-*score*. This further verifies the effectiveness of the multi-modal dual-stream network in processing complex data features and identifying samples of different categories. Future research can further optimize the model structure and try to verify its generality and robustness on more diverse data sets.

## 5. Conclusions

This paper studies the application of hybrid neural network models in epilepsy diagnosis using EEG signals. First, the complexity features of the EEG signal are extracted using various feature methods such as signal differential features, frequency domain amplitude spectrum and phase spectrum, etc., to form a two-dimensional time-series signal and two-dimensional spectrum features. In terms of network models, in order to extract the characteristics of EEG signals in multiple dimensions, three network structures are used, namely, one-dimensional convolution, two-dimensional convolution and lstm. Through the combination of multiple network structures, the multi-dimensional characteristics of EEG signals are trained. Finally, experiments were conducted on the public Bonn and New Delhi datasets to evaluate the effectiveness of the proposed model using indicators such as precision, recall, F1 score, etc. Finally, the test set results were analyzed using the confusion matrix and t-SNE. Our research results prove that the proposed network model achieved the best diagnostic effect in the experiment, with an *accuracy* of 0.9969, *precision* of 0.9944, *recall* of 1, and *F*1 *score* of 0.9972. Even after changing the data set, the hybrid mesh wheel still has the most stable classification performance and can achieve high accuracy in the diagnosis of epilepsy. This article provides a hybrid neural network model based on EEG for EEG signal epilepsy diagnosis, and uses a variety of feature extraction methods to provide a useful reference for the early detection and treatment of epilepsy.

## Figures and Tables

**Table 1:** Bonn EEG Dataset EEG Type.

	Healthy Control		Patients with Epilepsy		
Identifier	Z	O	N	F	S
State	Opened eyes	Closed eyes	Interictal period	Interictal period	Ictal period
Electrode position	Scalp	Scalp	Intracranial hippocampus	Intracranial lesion area	Intracranial lesion area

**Table 2:** Comparison and discussion with related studies.

Authors	Modeling Method	Dataset	Performance Metrics
Richhariya and Tanveer [15]	PCA, ICA and DWT	University of Bonn	*Accuracy* 99.0%
Li et al. [28]	Wavelet-based envelope analysis	University of Bonn	*Accuracy* 98.8%
Shen et al. [43]	Discrete wavelet transform and support vector machine	University of Bonn	*Accuracy* 97%, *sensitivity* 96.67%
Xu et al. [44]	1D CNN-LSTM	University of Bonn	*Accuracy* 99.39%, *Precision* 98.39%, *Recall* 98.79%, *F*1-*score* 98.59%
Proposed method	Multimodal dual-stream networks	University of Bonn	*Accuracy* 99.69%, *Precision* 99.44%, *Recall* 1%, *F*1-*score* 99.72%

## Data Availability

The publicly available datasets used in this paper are from the Bonn Epilepsy Dataset (https://repositori.upf.edu/handle/10230/42894), accessed on 1 November 2023. The Center for Neurology and Sleep at Hauz Khas, New Delhi (https://www.researchgate.net/publication/308719109_EEG_Epilepsy_Datasets), accessed on 1 November 2023.
